# Treatment of Cancer

**Published:** 1876-06

**Authors:** 


### TREATMENT OF CANCER.

Many persons who are troubled with cancer, or who imagine that they are—which is nearly as bad—have called on us or written to us, to inquire concerning the skill and professional standing of a physician to whom we have occasionally referred in our various articles on cancer. We desire to say that we had no intention of indorsing the doctor personally or professionally, for we never met him but once and then only for a few minutes, and we never heard of him previous to that time; but our impressions were favorable. What we intended to indorse is the plan of eradicating cancerous growths by means similar to those he employs. Some of the preparations employed are of a dangerous character, and in the hands of quacks have caused great suffering, and frequently hastened death. But a medicine properly compounded and applied by an intelligent physician, offers, in our opinion, the only promise of permanent relief. In reply to others, we will say, that we can not conveniently treat patients at their homes who reside far away from this city; but we will procure and forward to the attending physician the necessary medicines, with instructions for their use. All malignant tumors should be eradicated before the period of ulceration to secure the system against contamination, and the re-appearance of the disease.

